# Th1 and Th17 Cells in Tuberculosis: Protection, Pathology, and Biomarkers

**DOI:** 10.1155/2015/854507

**Published:** 2015-11-10

**Authors:** I. V. Lyadova, A. V. Panteleev

**Affiliations:** Immunology Department, Central Tuberculosis Research Institute, Yauza Alley 2, Moscow 107564, Russia

## Abstract

The outcome of *Mycobacterium tuberculosis* (*Mtb*) infection ranges from a complete pathogen clearance through asymptomatic latent infection (LTBI) to active tuberculosis (TB) disease. It is now understood that LTBI and active TB represent a continuous spectrum of states with different degrees of pathogen “activity,” host pathology, and immune reactivity. Therefore, it is important to differentiate LTBI and active TB and identify active TB stages. CD4^+^ T cells play critical role during *Mtb* infection by mediating protection, contributing to inflammation, and regulating immune response. Th1 and Th17 cells are the main effector CD4^+^ T cells during TB. Th1 cells have been shown to contribute to TB protection by secreting IFN-*γ* and activating antimycobacterial action in macrophages. Th17 induce neutrophilic inflammation, mediate tissue damage, and thus have been implicated in TB pathology. In recent years new findings have accumulated that alter our view on the role of Th1 and Th17 cells during *Mtb* infection. This review discusses these new results and how they can be implemented for TB diagnosis and monitoring.

## 1. Introduction

Tuberculosis (TB) is one of the most common infections in the world. Infection with* Mycobacterium tuberculosis (Mtb)* most often affects the lungs. The outcome of the pulmonary infection is divergent and can range from the complete pathogen clearance through asymptomatic latent infection (LTBI) to active TB disease.

LTBI has long been considered as a uniform state characterized by the immunological sensitization in the absence of clinical signs and symptoms of active TB disease. Recently it has become clear that LTBI includes a broad spectrum of states that differ by the degree of host immune resistance, inflammatory markers, and pathogen replication (i.e., by LTBI “activity”) [1].

The characteristics of the TB disease are in turn very diverse. They differ by the type of pathology developed in the lungs (e.g., tuberculoma, cavitary TB, and caseous pneumonia), the size of the affected lung tissue, the rate of disease progression, the characteristics of immune activation and inflammation, and* Mtb* load and replication (i.e., the presence, quantity, and replication activity of* Mtb* in the sputum). Overall, the outcome of* Mtb* infection is not a simple two-state distribution which includes LTBI and active TB but represents a continuous spectrum of states that differ by pathogen and host “activity,” have a different contagiousness, and require different treatment strategies. Therefore, it is important to accurately diagnose the stage and the status of* Mtb* infection.

It is generally assumed that the outcome of* Mtb* infection depends on natural variations in host immune response to mycobacteria, in particular on the type and extent of immune activation and inflammation. Immune activation and inflammatory reactions are essential for host protection against mycobacteria [2–6]. On the other hand, unrestricted immune responses are deleterious and may lead to the TB exacerbation [7, 8]. Overall, the interplay between immune activation, inflammation, and TB pathogenesis is complex and not completely understood. In spite of the complexity, associations between* Mtb* infection activity and some parameters of immune/inflammatory reactions have recently been described; several different immunological parameters have been suggested as markers of TB activity. Many of them are related to T helper cells (discussed below).

There are several populations (differentiation lineages) of T helper cells. The first two populations, Th1 and Th2, were described over 20 years ago [9]. Later, additional T helper subsets were identified, including Th17, Th22, Th9, and T_FH_. Among different populations of T helper cells, Th1 and Th17 are the main effector populations which mediate protection and pathology during TB. In this review we discuss the role for Th1 and Th17 cells in protective and inflammatory responses during* Mtb* infection and the prospective use of their markers for the evaluation of pulmonary TB activity.

## 2. Th1 Cells in TB Protection and Pathology

Distinct populations of T helper cells differ by the expressed cytokines and transcription factors and by their response to different classes of pathogens. Th1 cells produce IFN-*γ* and depend on the transcription factor T-bet. They are induced upon infection with intracellular pathogens and mediate protection mainly by activating macrophages which destroy intracellular pathogens. Th2 cells produce IL-4, IL-5, and IL-13, depend on the transcription factor GATA3, and fight extracellular parasites [9].* M. tuberculosis* is an intracellular pathogen and elicits Th1 response.

### 2.1. Th1 Induction and Differentiation

Effector Th1 cells differentiate from naïve lymphocytes. In general, the effector cell differentiation is driven by the signals received from TCR, costimulatory receptors, and cytokines. In the most simplified model, signals mediated by TCR and costimulatory receptors induce T cell activation and proliferation. Cytokine-derived signals are the main signals determining the differentiation lineage of antigen-activated T cells. Ligation of cytokines with their receptors activates the signal transducer and activator of transcription (STAT) factors. The STATs translocate to the nucleus and bind genes encoding lineage-specifying transcription factors (“master regulators”) and effector cytokines. These events determine the lineage of differentiating T helper cells [10].

The main Th1 inducing cytokines are IL-12 and IFN-*γ*. IL-12 is produced by antigen-presenting cells. Interaction of IL-12 with the IL-12 receptor, expressed on the surface of T cells, induces STAT4, which in turn induces T-bet, a master regulator of Th1 cells. T-bet binds directly to many Th1-specific genes (*Ifng*,* cxcr3*,* Il18r1*,* Il12rb2,* etc.) and positively regulates their expression [11]. T-bet also negatively regulates the expression of Th2- and Th17-specific genes and inhibits the differentiation of Th2 and Th17 cells. In the absence of T-bet, T helper cells differentiate towards Th2-like cells. STAT4 also directly binds to* Ifng* locus and stimulates IFN-*γ* production. STAT4 and T-bet cooperate to induce maximal IFN-*γ* production. In the absence of STAT4, T-bet does not induce optimal IFN-*γ* levels, and the combination of T-bet and STAT4 deficiency abolishes IFN-*γ* production [11–13].

IFN-*γ* acts by inducing STAT1. STAT1 synergizes with STAT4 in activating T-bet in T helper cells and also can directly activate Th1-associated genes [14, 15]. IL-4 and IL-10 inhibit Th1 cell differentiation and induce Th2 cells. Thus, generally, Th1 and Th2 are alternate and mutually exclusive lineages of T helper cells. In some conditions, however, Th1 and Th2 cells retain their plasticity, can transdifferentiate, and even coexpress Th1- and Th2-specific cytokines and transcription factors [16].

Cytokine milieu is the main factor that determines the lineage of T helper cell differentiation. However the differentiation is also affected by the dose and the strength of TCR agonistic ligand. It has been shown that stimulation with a high dose of TCR agonistic peptide or a strongly agonistic ligand favors generation of Th1 cells whereas a low dose or a weakly agonistic ligand favors Th2 cells [17]. Therefore, we may speculate that pathogen load, pathogen metabolic activity, and the stage of the disease affect the composition of the responding T helper population.

### 2.2. Th1 Cells and IFN-*γ* during TB

It has long been assumed that the central role of Th1 cells in the defense against TB is due to the ability of IFN-*γ* to activate macrophages and stimulate phagocytosis, phagosome maturation, production of reactive nitrogen intermediates, and antigen presentation [18–20]. This has been supported by many observations. In particular, mice deficient in CD4^+^ subset and Th1 type cytokines (i.e., IL-12p40, IFN-*γ*) succumb early to* Mtb* infection with high bacterial loads [21–23]. Similar effects are observed in mice with the defects in enzymes involved in the generation of host-bactericidal molecules, dependent on IFN-*γ* axis [24–27]. Humans with the mutations in molecules involved in Th1 immunity (i.e., the IL-12p40 subunit, the IL-12 receptor *β*1 chain, the IFN-*γ*-receptor ligand binding chain, and STAT1) exhibit extremely high susceptibility to infections induced by* Mtb*,* Bacillus Calmette-Guerin* (BCG), or environmental mycobacteria [28–30]. HIV-infected patients deficient in CD4^+^ cells have increased reactivation of latent* Mtb* infection and altered histopathological characteristics of TB disease (i.e., diffuse necrotic lesions instead of structured granulomas) [31]. These and other studies supported the concept that Th1 are prerequisite for TB defense and act by stimulating the antimycobacterial immunity through the Th1 → IFN-*γ* → macrophage activation →* Mtb* killing/restriction pathway. However some new unexpected results, that contradict this paradigm, are now emerging.

Several studies in mice reported lack of correlation between the degree of protection and the frequencies of* Mtb*-specific IFN-*γ* producing cells or IFN-*γ* levels [32–35]. In some studies, antigen-specific IFN-*γ* production by CD4^+^ T cells correlated with the decrease in* Mtb* load but did not reflect the strength of protection [34]. In other studies, Th1 mediated protection against* Mtb* replication was IFN-*γ* independent [36, 37]. Next, some authors reported that IFN-*γ* mediated protection by inhibiting a deleterious response of Th17 cells rather than by inhibiting* Mtb* replication [38]. Finally, in some experimental models CD4^+^ T cells were deleterious [39, 40].

For example, Scanga and coauthors reported that in the murine model of latent tuberculosis, depletion of CD4^+^ cells resulted in uncontrolled* Mtb* growth but it did not alter IFN-*γ* response [32]. Gallegos and colleagues [37] studied protective properties of ESAT-6 specific Th1, Th2, and Th17 cells derived from the wild type mice or mice deficient for factors associated with Th1 activity (e.g., IFN-*γ*, TNF-*α*). In an adoptive transfer model, Th1 cells were more protective than Th17 cells; Th2 cells were not exhibiting any protective activity against* Mtb* replication. Yet the ability of Th1 cells to mediate protection did not depend on IFN-*γ* or TNF-*α*.

Nandi and Behar adoptively transferred IFN-*γ* ^−/−^ memory CD4^+^ T cells into* Mtb*-infected recipients and assessed bacterial load and mice survival [38]. The latter is an integrated parameter of TB severity that largely depends on the extent of pathological inflammatory response. IFN-*γ* ^−/−^ memory CD4^+^ T cells retained their antimicrobial activity but failed to protect recipient mice against severe inflammation and death. It was suggested that IFN-*γ* acts by inhibiting IL-17 response and pathogenic neutrophil accumulation in the infected lungs, that is, that IFN-*γ* mediates protection by limiting host inflammatory response rather than by inducing macrophage antibacterial activity.

Finally, a deleterious role for the CD4^+^ T cells during TB has been demonstrated. Several studies documented that mice deficient in PD-1 and infected with* Mtb* exhibit unaltered or even increased CD4 and IFN-*γ* responses but die because of the severe infection characterized by the uncontrolled bacterial proliferation, increased lung tissue pathology, neutrophilic infiltration, and high lung expression of proinflammatory cytokines. Of note, CD4^+^ T cell depletion reduced production of IFN-*γ* and other proinflammatory cytokines and rescued PD-1^−/−^ mice from early mortality [39, 40]. Interestingly, resistance to viral infections is increased following PD-1 blockade [41], indicating that in TB an imbalanced T cell response may be more deleterious than during other infections.

Thus, one important aspect of TB immunity is that Th1 subset may mediate protection by mechanisms other than IFN-*γ* production or activation of host antibacterial activity and even may contribute to pathology.

Another puzzle comes from the studies in humans that investigated Th1/IFN-*γ* responses during LTBI and active TB.

LTBI is usually considered as a state of* Mtb* infection that develops due to an effective anti-TB immune defense. It is believed that the comparison of immune responses during LTBI and active TB may help with uncovering immunological correlates of protection. However, multiple studies of Th1/IFN-*γ* responses in humans have resulted in contradictory conclusions. Some studies reported that the antigen-driven secretion of IFN-*γ* by blood mononuclear cells is reduced in TB patients and normalized upon TB treatment [42–44]. On the other hand, frequencies of circulating IFN-*γ* producing cells are generally increased in TB patients and decrease following the treatment. In line with this, some studies showed increased plasma levels of IFN-*γ* in TB patients; these levels correlated with disease activity and normalized during the treatment [45]. Furthermore, patients with newly diagnosed TB were reported to have higher levels of IFN-*γ* compared to patients with chronic TB. Finally, it is now well documented that interferon-gamma-release assays (IGRAs) do not discriminate LTBI and active TB [46–48]. This indicates that the two groups do not differ consistently by the levels of* Mtb*-specific IFN-*γ* secretion (detected in Quantiferon-TB Gold-in tube assay, QFT) or by the frequencies of* Mtb*-specific IFN-*γ* producing cells (defined in T-SPOT-TB assay). Thus, no steady differences in IFN-*γ* responses have been identified so far between LTBI and active TB.

Considering TB patients, they are characterized by a great variability in the immune responses. It has been shown that IGRAs have suboptimal sensitivity for active TB, supposedly due to the immune suppression developed during severe disease. To verify this hypothesis, we have recently analysed whether the levels of IFN-*γ* secretion determined in QFT assay and the frequency of the antigen-specific IFN-*γ* producing cells determined by flow cytometry are associated with TB severity. For that, we checked the correlation of IFN-*γ* responses with the following characteristics of TB disease: the presence and the quantities of* Mtb* in the sputum, the forms of pulmonary pathology (i.e., tuberculoma, infiltrative TB, cavitary TB, caseous pneumonia), the degree of pulmonary destruction, TB extent, clinical disease severity, and hematologic abnormalities. We have found no significant association between these parameters and IFN-*γ* responses, which argues against the direct association between TB disease severity and the extent of IFN-*γ* response ([49], and our unpublished results).

### 2.3. Section Summary

Altogether, Th1 mediated IFN-*γ* response activates* Mtb* killing* in vitro* and contributes to the restriction of* Mtb* growth* in vivo* but also plays anti-inflammatory role during TB and can participate in TB exacerbation. Assays based on the evaluation of* Mtb*-specific IFN-*γ* responses are currently used to identify* Mtb* infection, but these assays do not allow discriminating LTBI and TB disease. Numerous comparisons of IFN-*γ* responses during active TB and LTBI have revealed no consistent patterns. There are several possible explanations for that: different anatomical distributions of effector Th1 cells during active TB and LTBI, functional exhaustion of effector Th1 cells during chronic TB disease, diversity of* Mtb* antigens expressed during LTBI and active TB, and a great variability of TB patients with regard to TB stage, host immune reactivity, and genetic and other factors. Irrespective of the underlying mechanisms, IFN-*γ* response seems to be unreliable for the evaluation of TB activity. However other markers of Th1 reactivity may be valuable and will be discussed below.

## 3. Th17 Cells in TB Defense and Pathology

Th17 were first described as a distinct population of the T helper cells that are controlled by the transcription factor ROR*γ*t and develop independent of T-bet, STAT4, GATA-3, and STAT6 transcription factors that are critical for the Th1 and Th2 development [50, 51]. The main effector cytokine of Th17 is IL-17; other cytokines are IL-22, IL-26, and GM-CSF [52–56].

The family of IL-17 cytokines consists of the six similar members, designated from IL-17A (often referred to as IL-17) to IL-17F. Th17 cells produce IL-17A and IL-17F. Both have similar biological activities and are the most thoroughly studied members of the IL-17 family. The family of IL-17 receptors includes five members (IL-17RA–IL17RE) that are expressed on dendritic cells (DC), macrophages, lymphocytes, epithelial cells, keratinocytes, and fibroblasts and allow different organs to respond to IL-17. IL-17A and IL-17F bind to IL-17RA [53, 57].

Th17 mediate pleiotropic activities that involve induction of proinflammatory genes (cytokines, chemokines, and metalloproteinases) and antimicrobial peptides, modulation of extracellular matrix, stimulation of granulopoiesis, recruitment, and activation of neutrophil granulocytes. Therefore, the main functions of Th17 cells are protection against extracellular pathogens and mediation of the inflammatory response, particularly during autoimmune and chronic inflammatory diseases.

### 3.1. Th17 Induction and Differentiation

The differentiation, expansion, and maintenance of Th17 cells depend on TGF-*β*, IL-1*β*, IL-6, IL-21, and IL-23. The role of these cytokines in the induction and maintenance/stabilization of Th17 cells is different in some species, in particular in mice and humans [53, 58–63]. In general, the differentiation of mouse Th17 cells depends on IL-6 and TGF-*β*. IL-23, IL-1*β*, and TNF-*α* maintain and amplify Th17 cells. Data on the differentiation requirements for human Th17 cells are contradictory. In most studies, human Th17 cells depended on different combinations of IL-1*β*, IL-6, and IL-23. TGF-*β* was dispensable or even inhibitory for their development (reviewed in detail in [64]). In both humans and mice different combinations of cytokines may result in the generation of different Th17 subsets (see below). IFN-*γ*, IL-12, IL-27, IL-4, and IL-2 inhibit Th17 differentiation [54, 58, 65–67].

At the molecular level, the generation of Th17 requires induction of ROR*γ*t, a key regulator of Th17 differentiation, which depends on the activation of the “pioneering factor” STAT3 [10]. IL-6, IL-21, and IL-23 activate STAT3, thus inducing the expression of ROR-*γ*t. Of note, IL-23 is able to induce the phosphorylation of both STAT3 and STAT4, but STAT3 phosphorylation is much stronger and biases T cell differentiation towards the formation of Th17 cells (the opposite is true for IL-12: it induces strong phosphorylation of STAT4 but relatively weak phosphorylation of STAT3 [53]). The role of TGF-*β* in Th17 differentiation is largely mediated through its capacity to inhibit the differentiation of Th1 cells (which suppress Th17) [68, 69]. It is assumed that, in the presence of TGF-*β*, IL-6 contributes to the generation of Th17 by inhibiting the differentiation of Treg. Indeed, TGF-*β* is critical for the generation of Treg. IL-6 inhibits Treg generation and thus in inflammatory conditions favors the generation of Th17; that is, it regulates Th17/Treg balance [70]. IL-21 was shown to upregulate its own expression and that of IL-21R and IL-23R. Besides ROR*γ*t, optimal generation of the Th17 cells also depends on other cofactors, such as IRF4, BATF, and RUNX1, which cooperate with ROR-*γ*t in Th17 differentiation [10].

### 3.2. Th17/IL-17 Biological Activity

Pleiotropic activities of Th17 cells involve activation and recruitment of neutrophils, stimulation of granulopoietic lineage of differentiation, and support of inflammation.

Neutrophil recruitment is mediated through the production of CXCL8 and GM-CSF [54, 71] and the induction of CXCL1, CXCL2, CXCL5, CXCL6, CXCL8, and G-CSF in tissue resident cells, in particular in human bronchial epithelial and venous endothelial cells [72]. CXCL1, CXCL2, CXCL5, CXCL6, and CXCL8 are neutrophil-attracting chemokines; GM-CSF and G-CSF stimulate granulopoiesis and granulocyte activation [73].

The ability of IL-17 to recruit neutrophils to mucosal sites has been demonstrated in a number of studies [74–77]. The role for IL-17 in modulating granulopoiesis is evident from the expansion of the neutrophil progenitors in the bone marrow and mature neutrophils in peripheral blood of the mice overexpressing IL-17 [78]. In the model of lung infection induced by* Klebsiella pneumoniae*, lack of IL-17 hampered stress-induced granulopoiesis suggesting that during infections intact IL-17 response may be required for the effective granulocyte generation [79]. In primary human bronchial epithelial cells Th17 induce the expression of mucins MUC5AC and MUC5B [80] and antimicrobial peptides (e.g., human beta-defensins) [81, 82]. The activity is mediated by IL-17 and IL-22 [83] and together with neutrophil recruitment serves to clear extracellular pathogens. Recent studies suggest that Th17 are also involved in host protection against some intracellular pathogens [84].

Th17/IL-17 mediated induction of chemokines and cytokines in tissue resident cells, as well as the recruitment of neutrophils, promote local inflammatory responses and may lead to the serious tissue damage. Accordingly, Th17 cells have been implicated in the pathogenesis of several autoimmune and chronic inflammatory diseases. It has been suggested that in autoimmunity and chronic inflammation, IL-17 synergizes with other proinflammatory cytokines abundantly produced in these pathological conditions, such as TNF-*α* and IL-1*β* [53]. Th17 cells may even coexpress IL-17 and TNF-*α*. It has been suggested that IL-17 rather sustains preexisting inflammation than induces it. This means that Th17 may be protective during acute infection and deleterious during chronic one [85]. This concept may be relevant to TB, as TB is accompanied by the abundant inflammatory responses.

### 3.3. Th17 Phenotype and Subpopulations

Naïve and antigen-experienced cells differ by the expression of their differentiation markers. Distinct lineages (populations) of T helper cells differ by the expression of chemokine receptors. Finally, some T cell lineages express lineage-specific markers. The majority of IL-17 producing cells express CD45RA^−^ phenotype of antigen-experienced cells [86]. Characteristic feature of IL-17 producing cells and their precursors is the expression of CD161, the lectin receptor, and the human ortholog of murine NK1.1 [87, 88].

With regard to chemokine receptor expression, the Th17 population is heterogeneous. It contains cells expressing lymphoid tissue homing receptor CCR7 (in a higher proportion than IFN-*γ* producing cells), B follicle homing receptor CXCR5, and nonlymphoid tissue homing receptors CCR4, CCR5, and CXCR6. Heterogeneous expression of these receptors by Th17 cells underlies their capacity to migrate to different sites [89].

Chemokine receptors CXCR3, CCR4, and CCR6 discriminate different lineages of T helper cells. Th1 cells are characterized by the expression of CXCR3, CXCR6, and CCR5; Th2 cells express CCR3, CCR4, and CCR8. The chemokine receptor associated with Th17 is CCR6, the receptor for CCL20 and defensins, mediating homing to skin and mucosal sites. Correlation between the expression of CCR6, the production of IL-17, and the expression of RORC (the human ortholog of mouse ROR*γ*t) was directly demonstrated, and Th17 cells were shown to express the CCR6^+^CCR4^hi^CXCR3^lo^ phenotype [90].

Further analysis, however, identified a population of CCR6^+^CCR4^lo^CXCR3^hi^ cells that coexpress Th17 (CCR6) and Th1 (CXCR3) associated receptors [91]. This phenotype is associated with the coexpression of two master regulators, ROR*γ*t and T-bet [10]. Functional analysis showed that CCR6^+^CCR4^lo^CXCR3^hi^ population contains cells producing only IFN-*γ*, only IL-17, and poly-functional cells able to produce both IFN-*γ* and IL-17. Due to the simultaneous production of IFN-*γ* and IL-17, the latter population was named Th1/Th17 [64, 90, 92–95].

Further heterogeneity of Th17 cells comes from their inflammatory properties. In mice, “pathogenic” and “nonpathogenic” populations of IL-17 producing cells have been described. Generation of pathogenic cells depended on IL-23. The cells produced IL-17, and their adoptive transfer caused experimental autoimmune encephalomyelitis. Nonpathogenic cells coexpressed IL-17A and IL-10, were able to suppress T cell proliferation and were called immune-suppressive or regulatory Th17 cells (rTh17) [96–99].

In humans, a population of Th17.1 cells similar to the “pathogenic” mouse Th17 cells has recently been described. The cells coexpressed CCR6 and CXCR3 and exhibited transient expression of c-kit and stable expression of the multidrug transporter MDR1. Transcriptionally and functionally Th17.1 cells resembled the pathogenic mouse Th17 cells; that is, they were highly sensitive to IL-23 stimulation and produced both Th17 and Th1 cytokines [91]. Similarly, in some studies, “pathogenic” Th17 cells in mice were also characterized by the coexpression of IL-17A, TNF-*α*, and IL-2. Altogether, the data raise questions on whether pathogenic potential of Th17 cells is associated with the CCR6^+^CXCR3^+^ population and whether it depends on IL-17 or on a combination of factors that these cells produce.

To summarize, the main phenotypic characteristic of IL-17 producing cells is the expression of CD161 and CCR6. Th17 are phenotypically and functionally heterogeneous and contain at least two subpopulations, Th17 and Th1/Th17, that differ by the expression of chemokine receptors (i.e., CCR4 and CXCR3) and effector cytokines (i.e., IL-17, IL-17 and IFN-*γ*, IL-17 and IL-10) and may play different roles in immune pathology and protection. Their exact role in these processes is yet to be determined.

### 3.4. Th17 and IL-17 in TB Protection and Pathology

The fact that Th17 cells mediate both antibacterial and proinflammatory responses suggests that their role during infection is complex. This is particularly true for TB, as the pathogenesis of TB critically depends on the extent of inflammation. Data on Th17 responses during TB are numerous but not uniform and quite likely depend on the model and the degree of inflammation.

Following vaccination, Th17 seem to contribute to memory response and protection. In mice immunized with BCG, IL-17 supported Th1 reactivity by downregulating IL-10 and upregulating IL-12 production in dendritic cells [100, 101]. Following* Mtb* challenge of BCG vaccinated mice, Th17 induced chemokines, recruited CD4^+^ T cells to the site of infection, favored granuloma formation, and accelerated pathogen clearance [102]. In humans, generation of Th17 and Th1/Th17 cells in response to a novel TB vaccine, the modified vaccinia virus Ankara expressing antigen 85A, was documented [103]. Interestingly, BCG vaccination induces different levels of IL-17 in genetically different mice. Garcia-Pelayo and coauthors [35] have demonstrated that BALB/c mice produce more IFN-*γ* and IL-17 and less IL-10 in response to BCG as compared to C57BL/6 mice. This suggests that (i) the extent of Th17 reactivity is affected by the host genetic factors; (ii) the pattern of Th1/Th2/Th17 responses may differ during TB infection and following BCG vaccination.

Data on the role for Th17 during primary* Mtb* infection are conflicting.

In mice challenged via aerosol route with a low dose of laboratory* Mtb* strain, IL-17 was dispensable for primary immunity [104]. On the other hand, Th17/IL-17 responses were involved in the protection against highly virulent* Mtb* isolate, HN878. Gopal and coauthors [105] reported that mice challenged with HN878 exhibited elevated IL-17/Th17 responses as compared to mice challenged with laboratory adapted* Mtb* strain. IL-17^−/−^ mice infected with HN878 had elevated lung bacterial burden, diminished chemokine response (CXL13, in particular), defective formation of ectopic lymphoid follicles, and hampered colocalization of T-lymphocytes and macrophages [105]. The involvement of Th17 in TB protection is also supported by partial inhibition of the* Mtb* growth following the adoptive transfer of Th17 cells [37].

The role for Th17/IL-17 responses in TB protection in humans was mainly studied by comparing these responses in TB patients and healthy individuals. The results are contradictory. Some authors reported similar levels of IL-17 in the blood and bronchoalveolar fluids (BAL) of TB patients and healthy controls [106]. In other studies, the frequencies of blood IL-17 producing cells were reduced in TB patients suggesting that Th17 contribute to the protection [107]. This idea is supported by the observation showing that the low level of IL-17 in serum is associated with high mortality of TB patients [108].

Other studies, however, suggest the involvement of Th17 in TB pathology. Jurado and coauthors reported an augmented Th17 response in TB patients. The major source of IL-17 was represented by IFN-*γ* ^+^IL-17^+^ CD4^+^ T cells, and their proportion directly correlated with the clinical parameters associated with the disease severity [109]. In line with this, Basile and others have associated augmented Th17 response with persistent and high antigen load and pathogen drug resistance [110]. It should be noted that IL-17 has been implicated in neutrophil recruitment and stress-induced granulopoiesis. Neutrophils have been suggested to play a pathogenic role and exacerbate TB disease [7, 8, 111–113]. Recent studies performed in a mouse model have suggested that immature myeloid cells are strong correlates of severe TB [114–116]. Thus, the involvement of Th17/IL-17 responses in the pathogenesis of severe TB is not surprising.

An important question is the interaction/s between Th17 and Th1 cells and their relative roles during active TB and LTBI. As discussed above, Th1 cells and IFN-*γ* suppress the generation of Th17. On the other hand, Th17 do not inhibit the generation of Th1* in vitro* and may even favor Th1 response* in vivo*. The relationships between Th1 and Th17 cells during TB infection are complex and not well understood. Comparative analysis of Th1 and Th17 responses was performed in several studies and the results are contradicting. Marín and coauthors [117] studied the frequencies of IL-17 and IFN-*γ* producing cells in patients with active TB, LTBI, and noninfected control donors. The frequency of IFN-*γ* producing cells was elevated in LTBI, and IL-17 producing cells were more frequent in TB patients. The authors concluded that active TB biases the protective Th1 profile toward the pathological Th17 response. Another study compared cytokine levels in tuberculin skin test (TST) positive and TST negative individuals in TB endemic area. Th1 and Th2 cytokines were not different between the two groups; IL-17, IL-23, and ROR*γ*t expressions were downregulated in TST positive individuals. The authors suggested that lack of Th17 cells predisposes to latent infection [118]. However this study did not investigate active TB. Li and coauthors evaluated studies of Th1 and Th17 responses in TB patients [119]. Of 226 studies, nine met their criteria and were selected for the analysis. Their systematic review showed that in TB patients the levels of IL-17 and IFN-*γ* were low; during LTBI IL-17 and IFN-*γ* levels were generally high compared to active TB. In contrast to TB disease, BCG vaccination in children induced high level of IL-17 and IFN-*γ*. The authors concluded that after BCG vaccination and during* Mtb* infection IL-17 acts as an effector molecule similar to IFN-*γ* and together with IFN-*γ* contributes to TB protection.

An interesting question is which cells represent the major source of IL-17 during TB. As discussed above, a population of Th1/Th17 cells coexpressing IFN-*γ* and IL-17 exists. Th17 may acquire expression of T-bet and IFN-*γ* and even Foxp3 during their development; that is, they exhibit substantial plasticity [10, 16, 120]. Whether “multifunctional” Th17 cells differ from “classical” Th17 cells in their pathogenicity is not completely clear. As noted above, in some pathological conditions (e.g., in the gut of Crohn's disease patients), cells coexpressing Th17 and Th1 cytokines are pathogenic. Similarly, in TB patients the accumulation of IFN-*γ* ^+^IL-17^+^ cells correlated with the disease severity [109]. Arlehamn and coauthors [121] have demonstrated that TB-specific memory T cells are predominantly present within the CCR6^+^CXCR3^+^CCR4^low^ population and that this population is significantly increased in LTBI donors compared to healthy controls. The CCR6^+^CXCR3^+^CCR4^low^ population is known to contain Th1/Th17 cells. However, the authors did not detect IL-17 production in TB-specific CCR6^+^CXCR3^+^CCR4^−^ cells upon their* ex vivo* antigenic stimulation. Note that in response to other antigens (e.g.,* Candida albicans*) CCR6^+^CXCR3^+^CCR4^low^ cells readily produce IL-17 [90]. Thus, the accumulation of* Mtb*-specific CCR6^+^CXCR3^+^CCR4^−^ IL-17^−^ cells may represent a characteristic feature of* Mtb* infection. It would be interesting to study if these cells also accumulate during active TB or if they specifically mark LTBI.

Another question concerns Th17 responses that develop locally in the lungs. The characteristics of local Th17 responses could probably shed a light on the role which these cells play in TB protection/pathology, but this aspect has not been studied in detail yet. In one study, IL-17 was not abundant in pleural or pericardial fluid of TB patients; IL-17 expression by mycobacteria-specific disease site T cells was not detected in healthy* Mtb*-infected persons, or patients with TB pericarditis, allowing the authors to conclude that IL-17 does not play a major role at established TB disease sites in humans [122].

### 3.5. Section Summary

To summarize, a number of studies suggest that IL-17 producing T cells are efficiently generated following vaccination and involved in the memory response to subsequent* Mtb* challenge. The role for these cells during primary* Mtb* infection is less clear. In many studies, the extent of Th17 response during TB infection was low, which may be interpreted as a defect in the protective response, but also as a dispensable role for Th17/IL-17 in TB protection and pathology. However, other studies have associated Th17/IL-17 with TB pathology and progression. It is possible that Th17 cells may play different roles at subsequent stages of TB infection providing protection at early disease stage but inducing pathology at advanced TB. Next, IL-17 producing T helper cells contain at least two different populations, Th17 and Th1/Th17. Their role in TB protection and pathology may differ and has not been evaluated separately as were the peculiarities of local Th17 responses. It should be noted that experimental data on the role for Th17 cells during* Mtb* infection should be interpreted with caution. Indeed, unlike Th1 and Th2 cells, Th17 cells differ between mice and humans. In particular, mouse and human Th17 cells depend on different sets of cytokines, and the efficacy of their generation during* Mtb* infection in mice and humans may differ. Next, mouse models of TB infection do not fully reproduce pulmonary TB in humans by the type of pulmonary pathology and granuloma formation. Thus, in mice and humans cells involved in granuloma formation may have a different impact in local immune responses. Finally, clinical* Mtb* isolates and laboratory* Mtb* strains induce a different extent of Th17 response.

It should also be noted that discriminating between protective and pathological responses during infectious diseases is always extremely difficult, as the same cells may be simultaneously involved in both processes. However some parameters of cellular responses (regardless of their protective or pathological impact) may be strongly associated with the disease severity/activity and thus may be used as biomarkers for disease evaluation and monitoring. Whether biomarkers of Th1 and Th17 responses may be used to assess activity of* Mtb* infection is discussed in the next section.

## 4. T Cell Associated Biomarkers of TB Activity

As discussed in the introduction, evaluation of TB activity is an important diagnostic goal, both to discriminate between LTBI and active TB and to monitor infection activity at the different stages of TB disease. Whether and which parameters of the immune response may be used as markers of TB activity are an open question.

### 4.1. IFN-*γ*

Speaking about IFN-*γ* response, which has been studied most extensively, the relations between its intensity and* Mtb* infection activity are extremely complex. In the most simplified model, the efficacy of IFN-*γ* response depends on genetic and/or other* Mtb* infection-independent factors (e.g., nutrition, stress, the use of immunosuppressive drugs, and chronic infections) and this affects the outcome of* Mtb* infection. In this model, the lower the IFN-*γ* response is, the higher the TB activity would be. On the other hand, all immune responses, including IFN-*γ*, are pathogen-driven and thus the more active the infection is, the higher the immune response should be. However, chronic infection and persistent antigenic stimulation induce regulatory loops and T cell exhaustion, affecting the relationships between infection activity and IFN-*γ* production. The real situation is even more complex because IFN-*γ* is produced by different immune cells, including innate and adaptive cells, which may react to the infection differently. Finally, the IFN-*γ* producing cells accumulate preferentially at the sites of infection, so their reduced (or increased) response in peripheral blood may be associated with increased (or diminished) local responses.

This complexity explains why a simple measure of IFN-*γ* response does not allow to evaluate TB activity. Indeed, the levels of antigen-specific IFN-*γ* production by mononuclear cells or the frequencies of* Mtb*-specific IFN-*γ* producing cells, evaluated in IGRAs or by flow cytometry, do not discriminate between LTBI and active TB. Also, the levels of IFN-*γ* response are not associated with the disease severity in TB patients ([47–49, 123]; our unpublished observations). However, some other parameters of Th1 response may be used as TB biomarkers.

### 4.2. Markers of Th1 Differentiation and Activation

Several studies have demonstrated that phenotypic markers of T cell activation and differentiation are promising biomarkers of TB activity. Following antigen-driven differentiation, T lymphocytes pass through several stages (early, late, and terminally differentiated effector cells). Each stage is characterized by the set of markers expressed on the surface of T lymphocytes. As the differentiation process depends on antigenic stimulation, markers of T cell differentiation may serve as indicators of TB activity. Among such markers CD27 seems to be best studied.

CD27 is a member of TNF receptor superfamily. It is constitutively expressed by the naive T cells and early effector lymphocytes but downregulated at the late stages of effector cell differentiation. Therefore, late effector lymphocytes exhibit low to no CD27 expression [124–130].

In mice, the CD27^low^ phenotype has been linked to efficient IFN-*γ* production and lung-homing properties of the effector CD4^+^ T cells [130, 131]. Moreover, it was demonstrated that CD27^low^ effector CD4^+^ T cells can differentiate from CD27^hi^ effector precursors directly in the lungs infected with* Mtb* [131]. The data suggest that CD27^low^ population may serve as a measure of pulmonary TB activity. This, indeed, was demonstrated in several studies performed by several scientific teams.

In the pilot study, Streitz and others [132] examined the percentages of CD27^−^ cells within the population of blood* Mtb*-reactive CD4^+^ T cells that were identified as CD4^+^ cells producing IFN-*γ* in response to the tuberculin stimulation. High (>49%) percentage of CD27^−^ (IFN-*γ* ^+^CD4^+^) cells discriminated patients with smear and/or culture positive pulmonary TB from patients with smear/culture negative TB and LTBI with 100% sensitivity and 85.7% specificity.

The potential of “CD27/IFN-*γ*” approach to discriminate active TB and LTBI was also reported by other authors [49, 133–135]. Some studies found an association between the accumulation of blood CD27^−^ *Mtb*-reactive CD4^+^ T cells and bacillary load in TB patients [133]. In our study, the frequencies of CD27^−^IFN-*γ* ^+^ CD4^+^ cells strongly correlated with a degree of pulmonary destruction. Evaluation of blood CD27^−^IFN-*γ* ^+^ CD4^+^ cells allowed not only to separate active TB and LTBI, but also to assess the degree of pulmonary destruction, that is, the activity of pathological process ongoing in the lung tissue during TB and following the treatment [49]. Schuetz and coauthors modified this approach and extended it to HIV^+^ patients. The authors reported that HIV^+^TB^−^ patients have higher proportion of CD27^−^IFN-*γ* ^+^ CD4^+^ cells than HIV^−^TB^−^ patients and suggested that in HIV-infected individuals the accumulation of* Mtb*-reactive CD27^−^ CD4^+^ cells mirrors a degree of* Mtb* replication and may help to identify subclinical* Mtb* infection [134]. The same group has recently extended their study made in adults to children. The assay the authors called “TAM-TB” (for “T cell activation marker-tuberculosis”) detected culture confirmed TB cases in children with 83.3% sensitivity and 96.8% specificity [135]. Petruccioli and coauthors compared diagnostic accuracy of different variations of “CD27/IFN-*γ*” approach and concluded that CD27 expression is a robust biomarker for discriminating between TB stages [136].

To summarize, the “CD27/IFN-*γ*” approach has been validated in several studies made in few independent laboratories. In addition, it relies on the mechanisms that are generally well understood and involve promoted local generation of CD27^−^ cells in* Mtb*-infected lungs and their facilitated emigration from the lungs to the circulation system when lung tissue is destructed [49, 131].

Another marker which can be used to differentiate active TB and LTBI is CD57. CD57, the human natural killer-1 (HNK-1) glycoprotein, marks terminally differentiated, proliferatively incompetent cells [137]. Lee and others demonstrated that increased frequencies of CD57^+^ cells among ESAT-6/CFP10-responding CD4^+^ T cells differentiate active TB from LTBI [138].

In contrast to differentiation, which is an irreversible process, the activation of T cells is a temporal state that comes as a result of T cell encounter with a cognate antigen. T cell activation is characterized by upregulation of several surface molecules. Some of them, in particular, CD38 and HLA-DR, have been associated with active TB. CD38 is a transmembrane glycoprotein that has ectoenzymatic properties and catalyzes the synthesis and hydrolysis of NAD and cyclin ADP-ribose. HLA-DR is a member of MHC class II molecules involved in antigen presentation. Both markers are upregulated by antigen-responding T cells. Several recent studies suggested that increased frequencies of CD38^+^ and HLA-DR^+^ *Mtb*-reactive (IFN-*γ* producing) blood CD4 T cells allow accurately separating active TB from LTBI and even predicting the time of sputum conversion [123, 139].

### 4.3. Multifunctional T Cells

Besides IFN-*γ*, Th1 cells produce other cytokines, such as TNF-*α* and IL-2. Th1/Th17 cells coexpress IFN-*γ* and IL-17. Thus, effector T cells may exhibit multifunctional activities. It has been suggested that there is an association between T cell multifunctionality and TB activity.

Harari and coauthors reported that TB patients had reduced frequencies of multifunctional (IFN-*γ* ^+^TNF-*α* ^+^IL-2^+^) cells and increased proportion of single-positive TNF-*α* producing cells (TNF-*α* ^+^IFN-*γ* ^−^IL-2^−^) as compared to LTBI patients [140]. The authors also showed that TB patients are characterized by a higher content of* Mtb*-specific CD8^+^ T cells [141]. Combined determination of the frequencies of single TNF-*α* ^+^ cells and* Mtb*-specific CD8 T cells predicted active TB with 86.5% specificity and 81.1% sensitivity [142]. Several other groups also reported reduced frequencies of multifunctional cells in TB patients and their recovery following TB treatment [143].

In contrast, some other studies associated active TB with increased proportions of multifunctional cells. Caccamo and coauthors reported that patients with active TB had high frequencies of multifunctional (IFN-*γ* ^+^TNF-*α* ^+^IL-2^+^) lymphocytes and these frequencies decreased following the treatment; during LTBI single IFN-*γ* and double IFN-*γ* ^+^IL-2^+^ *Mtb*-responding cells predominated [144]. In another study, active TB was associated with bifunctional (IFN-*γ* ^+^TNF*α* ^+^) CD4^+^ T cells [145].

Chesov and coauthors using a novel dual cytokine detecting fluorescence-linked immunospot (FluoroSpot) assay found that the number of single-positive IL2^−^IFN-*γ* ^+^ cells was higher in patients with active TB compared with past TB and LTBI. However, there was the overlap in cytokine responses, which precluded distinction between the cohorts and suggested that the combined analysis of IL-2 and IFN-*γ* producing cells does not allow to separate different states of* Mtb* infection in clinical practice [146].

In HIV-infected individuals, analyses of T cell cytokine profile also gave contradictory results. Some studies reported higher frequencies of single functional TNF-*α*-only-secreting T cells in HIV^+^TB^+^ patients [147], while others found comparable cytokine profiles of* Mtb*-reactive CD4^+^ T cells in HIV-infected patients with and without active TB [148].

A separate population of multifunctional cells is represented by Th1/Th17 lymphocytes expressing CCR6^+^CXCR3^+^ phenotype. Recently, Sette group described a remarkable expansion of CCR6^+^CXCR3^+^ cells in LTBI [95]. The cells were multifunctional as they produced IFN-*γ*, TNF-*α*, and IL-2 upon stimulation with TB-derived epitopes, but the cells did not produce IL-17. Besides that, the authors did not analyze CCR6^+^CXCR3^+^ cells during active TB. Thus, whether Th1/Th17 and/or CCR6^+^CXCR3^+^ cells can serve as markers of LTBI or TB disease is still an open question.

Overall, current data on whether and how multifunctionality of effector T cells is associated with TB activity are contradictory. It also remains unclear whether multifunctional cells are more protective or more pathological compared to single TNF-*α* or IFN-*γ* producers, whether different cytokine profiles mirror different degrees of T cell maturation, and whether they may serve as reliable biomarkers of TB activity. Thus, more studies are required for the further implementation of this approach.

## 5. Conclusions

During the past decade a number of attempts have been made to discover reliable TB biomarkers. In the field of TB, studies are generally aimed to identify biomarkers for (i) rapid TB diagnosis, including diagnosis of sputum-negative TB cases; (ii) differentiation of active TB and LTBI; (iii) monitoring TB activity; (iv) prediction of LTBI conversion to active TB; (v) assessment of treatment efficacy.

In this review we have mainly focused on T cell associated markers that have been suggested to discriminate active TB and LTBI. Analysis of these biomarkers shows that most of them are at Phase I of discovery according to the classification of the National Institutes of Health, need to be validated in several independent laboratories, and undergo other evaluations. It should also be noted that the most promising T cell associated biomarkers described above are detected by means of flow cytometry. Their implementation in clinical laboratories may be challenging and will require assay simplification. However if the value of the developed approaches is confirmed, this might be possible.

It is understood that immunological approaches for TB diagnosis and monitoring are inferior to the direct identification of* Mtb*. However immunological approaches may be of great value for detecting paucibacillary and paediatric tuberculosis cases, TB screening, and monitoring treatment efficacy beyond sputum conversion. Uncovering immunological TB biomarkers will also allow a better understanding of TB pathogenesis.
